# The “teach-back” method improves surgical informed consent and shared decision-making: a proof of concept study

**DOI:** 10.1186/s13037-022-00342-9

**Published:** 2022-10-28

**Authors:** Kevin D. Seely, Jordan A. Higgs, Lindsey Butts, Jason M. Roe, Colton B. Merrill, Isain Zapata, Andrew Nigh

**Affiliations:** 1 Division of Clinical Medicine and Surgery, College of Osteopathic Medicine, Rocky Vista University, UT 84765 Ivins, UT USA; 2 Department of Research and Development, Rocky Vista University, Parker, CO USA

**Keywords:** Surgery, informed consent, teach-back method, surgeon trust, communication, patient education, patient safety

## Abstract

**Introduction:**

The teach-back method is a communication tool that can improve patient safety and shared decision-making. Its utility in patient care has been studied extensively in many areas of clinical medicine. However, the literature on teach-back in surgical patient education and informed consent is limited, and few studies have been conducted to test its impact on perioperative patient interactions. The objective of this study was to evaluate if the teach-back method can improve informed consent and surgeon trust. An assessment of the time required to be implemented was also evaluated.

**Methods:**

A standardized interaction role-playing a pre-operative informed consent discussion was designed. Laparoscopic cholecystectomy was selected as the proposed procedure. Standardized patients were split into two groups: teach-back and a control group. The control group was delivered a script that discloses the risks and benefits of laparoscopic cholecystectomy followed by a concluding prompt for any questions. The teach-back group was presented the same script followed by the teach-back method. Interactions were timed and patients completed a quiz assessing their knowledge of the risks and benefits and a survey assessing subjective perceptions about the interaction. Statistical analysis through Generalized Linear Models (GLMs) was used to compare visit length, performance on the comprehension quiz, and subjective surgeon trust perceptions.

**Results:**

34 participants completed the scenario, the comprehension quiz, and the survey (n = 34). Analysis of the subjective evaluation of the physician and encounter was significant for increased physician trust (p = 0.0457). The intervention group performed higher on the knowledge check by an average of one point when compared to the control group (p = 0.0479). The visits with intervention took an average of 2.45 min longer than the control group visits (p = 0.0014). People who had the actual procedure in the past (evaluated as a confounder) were not significantly more likely to display the same effect as the teach-back method, suggesting that the knowledge and trust gained were not based on previous experiences with the procedure.

**Conclusion:**

When employed correctly by surgeons in the perioperative setting, the teach-back method enhances shared decision-making, comprehension, and surgeon trust. Incorporating the teach-back method into risk and benefit disclosures effectively informs and more fully engages patients in the informed consent process. Notably, the added benefits from using teach-back can be obtained without a burdensome increase in the length of visit.

## Introduction

The physician-patient relationship depends on a foundation of trust and open communication. Ineffective communication within the physician-patient relationship may lead to detrimental or even harmful outcomes [1, 2]. Good communication is a teachable skill that depends on a variety of aspects, including not only the words spoken, but also context, setting, body language, patient expectations, physician workload and state of wellness, self-interest, and communication tools such as the teach-back method [3]. The objectives of physician-patient communication include facilitating an environment of mutual understanding, building a connection, enabling the flow of information, and including patients in decision-making [4]. It also needs to be made sure that the important points of key information are received and understood.

**Fig. 1:**
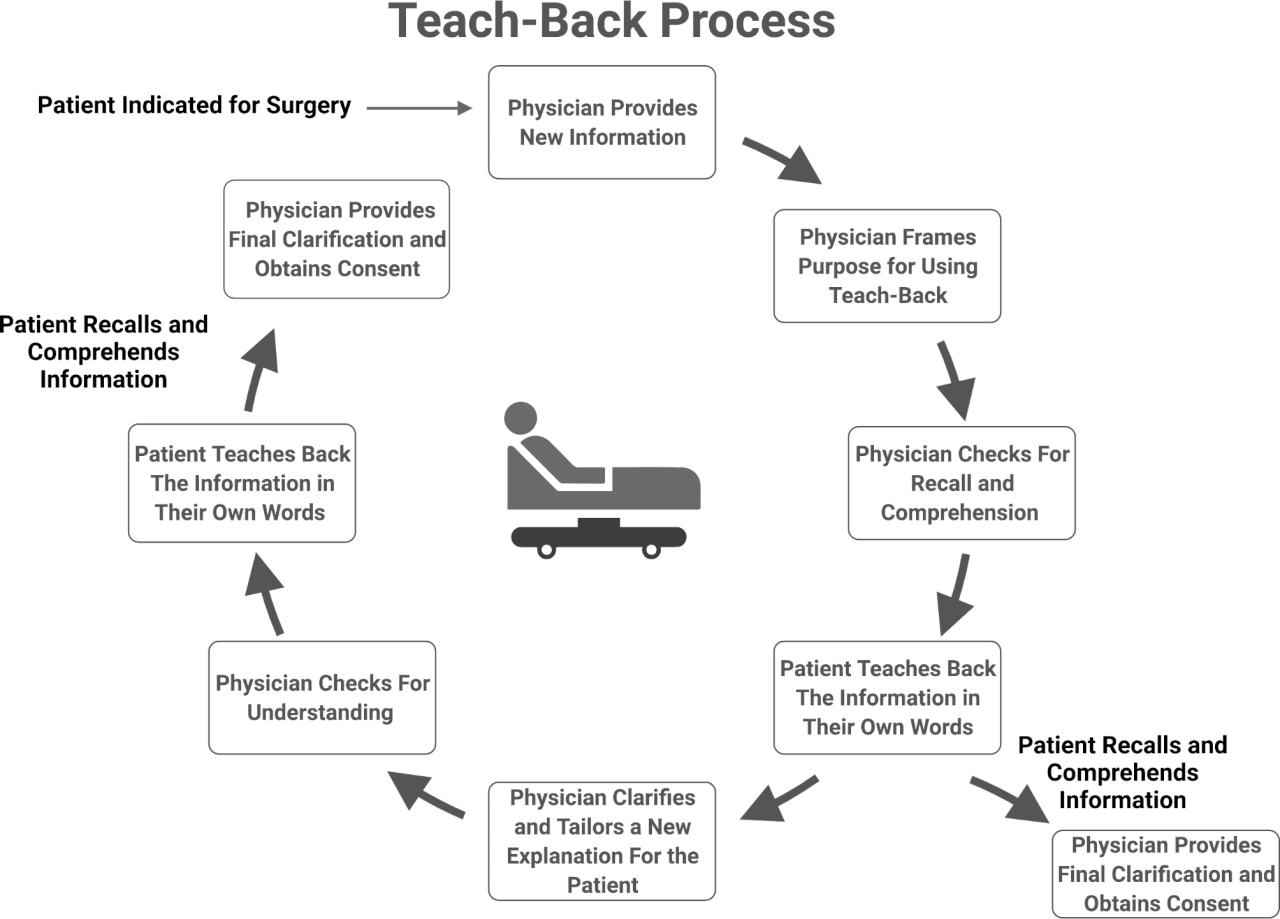
Teach-back is a dynamic, interactive, and patient-centered process that may require multiple repeated sequential explanations, checks for comprehension, and clarifications. Figure used with permission from Seely et al. [13]

The teach-back method allows the physician to communicate openly, ask patient-specific questions, and identify and resolve any misunderstandings in real-time, thus improving the comprehension of information [5, 6]. The method consists of multiple steps involving the clinician introducing new information, assessing the recall of the patient by asking them to repeat what they understood, and clarifying and rephrasing the information catering to the patient’s level of understanding. The physician then reassesses the patient’s understanding. It has been suggested that this cycle be repeated as many times as necessary for comprehension by the patient [7–9] (Fig. 1).

The teach-back method has been shown to improve communication and enhance the relationship between patients and their healthcare providers to meet many of the aims of effective communication. However, current literature on the teach-back method’s application to the pre-surgical informed consent process is limited [10–13]. Therefore, the objective of this proof-of-concept study was to provide foundational evidence to determine the beneficial effect of the teach-back method for pre-surgical informed consent based on a clear actionable definition and formula.

## Methods

### Participants

Adult volunteers were recruited and consented for participation in a standardized interaction role-playing a preoperative informed consent discussion. The study was vetted by the Institutional Review Board. The participants were split into two equal groups which were established at random: a teach-back group and a control group. Randomization was achieved through patients drawing a number and a letter out of a stack of index cards. Informed consent was obtained from each patient before the scenarios were conducted. Student doctors acted as the surgeons and wore professional dress for the interactions and the interactions were held in authentic exam rooms located on a medical school campus in St. George, Utah. Inclusion criteria: All participants were included as long as they were not members of the medical school campus teaching faculty or members of the student population. Exclusion criteria: No individuals were excluded provided they have met the inclusion criteria. Exclusion and inclusion criteria were designed to replicate the average population stratification seen in clinics.

### Scenario

It was decided to use biliary colic and laparoscopic cholecystectomy as the suggested diagnosis and treatment in the scenario because of its elective acuity level and well-defined alternative. The entire interaction was set up to simulate a real office scenario. The patients were scheduled and instructed to arrive at the standardized patient simulation center on a medical school campus. They were greeted in a waiting area outside of the exam rooms. Before the interaction, patients were instructed to act as they normally would at a doctor’s visit, i.e., as themselves. The opening instructions provided to each participant were as follows, “Welcome to this research study. Thank you for your participation. The student doctors are not being graded. Throughout the visit, please do not focus on the administration skills of your administrator, but rather, on the content of the discussion and your genuine reaction to the discussion. Upon completion of this experience, you will complete a quiz and a survey. The discussion will be done in the context of a scenario that will be provided to you. When the student asks you if you consent to the procedure, say yes. The answer to the first survey question is the number on the piece of paper you have selected.”

The patient-actors were asked to put themselves in the context of the patient scenario provided to them, which was the same for all participants in order to maximize standardization and minimize the demographic variable effect. The scenario was then provided to the participants, “You are a 35-year-old with one attack of right upper quadrant pain, 4/10, after eating some pizza. The attack came on in a spontaneous fashion 30 minutes after eating. The pain was sharp, intermittent, and associated with slight nausea, no emesis. You have had one previous similar episode. No alleviating or aggravating factors, except eating pizza. The pain resolved after two hours and one acetaminophen tablet: no dark urine, light stools, or signs of jaundice. The rest of the histories and physical examinations are unremarkable. Gallbladder ultrasound reveals multiple gallstones, no symptoms of acute cholecystitis, and no signs of biliary obstruction. You are here today to speak with a general surgeon about having your gallbladder removed.”

The patients were escorted to the exam room where they waited for the surgeon to enter. The timer began when the surgeon entered the room and ended when the surgeon exited the room. The conversation began with an unstandardized greeting, followed by the standardized portion which consisted of a composed script that was the same for both groups and conclusions that were uniquely designed for the control group and the teach-back group.

The script outlining the risks and benefits of laparoscopic cholecystectomy was recited to the control group, followed by a conclusion that consisted only of an invitation to ask any questions. The teach-back group was presented with the same script followed by the teach-back method (Table 1).

**Table 1:** Control group conclusion vs. teach-back group conclusion

Control Conclusion 1. “What questions do you have? 2. Answer questions 3. “Do you consent to the procedure?”
Teach-back Conclusion 1. “I want to make sure I explained this to you well, to do so, in your own words will you please state back to me what you understand about the risks, benefits, and alternatives to having gallbladder removal surgery?” 2. Listen to the patient 3. “Thank you for your response. I would like to clarify some key points.” 4. Expound upon key points and make specific corrections 5. “Again, I want to make sure I explained this well. Now that we have clarified some key points, would you again repeat back what you understand about the risks, benefits, and alternatives to having gallbladder removal surgery?” 6. Listen to the patient 7. Thank the patient and clear up any final misunderstandings. 8. “Do you consent to the procedure?”

Interactions were also timed using mobile phone timer apps to compare the length required to perform teach back to the time required for the control scenarios. After the interaction, patients scanned a QR code on their cellular devices that linked to Qualtrics where they completed a quiz testing their knowledge of the risks and benefits of the explained procedure and a survey assessing their subjective perceptions of the interaction (Tables 2 and 3).

**Table 2:** Post-interaction Comprehension Quiz

Indicate whether the following statements are true or false
1. The major risks of gallbladder removal surgery are bleeding and infection 2. I am required to follow a low-fat diet after my surgery that consists of soft foods and liquid 3. There are alternative approaches to managing gallstones, including not having surgery and taking medication for life 4. In the case of inflammation or the surgeon’s inability to visualize the gallbladder, a large single incision may be used to open the abdomen which would require a longer hospital stay and a more difficult recovery 5. I must keep the incisions dry for 72 h 6. Five small incisions will be made, leaving small scars of no more than 1 inch 7. If your surgery is scheduled for 9:00 am, you should arrive at the hospital at 8:00 am for registration and preparation 8. You can usually resume normal activities 3 days after surgery 9. Diarrhea or altered stool consistency or color is common after gallbladder removal surgery 10. You will have some pain following your surgery, which will be manageable.

**Table 3:** Post-interaction Subjective Perceptions Survey

Please indicate whether you strongly disagree, disagree, agree, or strongly agree
1. After speaking with the surgeon, I feel that I comprehend the risks and benefits of my surgery enough to confidently make a decision 2. I was adequately informed about alternatives to surgery 3. The surgeon resolved my misunderstandings, concerns, or questions 4. I trust this surgeon more after our discussion of the risks and benefits of surgery 5. The physician helped me to make a well-informed decision about my healthcare

To measure the effect of previous personal experience with the procedure, participants were also prompted to optionally indicate whether or not they themselves have actually had a laparoscopic cholecystectomy in the past. This was to address previous knowledge of the procedure as a confounder.

### Statistical analysis

Quiz and survey responses were evaluated to assess the effect of the teach-back method. The parameters evaluated were visit length (converted to seconds), performance on the comprehension quiz (total correct score), and individual surgeon trust perceptions (Likert scale average). Preliminary power analysis determined that a sample of 34 participants (17 per group) was required to detect a difference of 0.50 at 80% power and 95% confidence. This power calculation was performed using G*Power v.3.1.9.4 [14]. To do this assessment, all data was evaluated through Generalized Linear Models (GLMs).

Normality assumptions were checked using the residual plots, none of the parameters evaluated displayed any normality assumption violations. Each of the parameters assessed was evaluated for association against the treatment group (teach-back method vs. control) and the confounder (previous knowledge of the procedure). We hypothesized that by utilization of the teach-back method compared to a scripted description of procedure-specific risks and benefits, the group that received the teach-back intervention would demonstrate better retention of information and develop a measurably greater trust in the surgeon. To evaluate our hypotheses, the goal was to detect association to the teach-back method but not the confounder. All statistical analyses and descriptive statistics were performed in SAS/STAT v. 9.4 (SAS Institute, Inc., Cary, NC). Significant differences were declared at P ≤ 0.05.

## Results

A total of thirty-four volunteers participated in this study (n = 34). In this study, no demographic data was collected. The average total score for the knowledge quiz was 7.87 (SD = 1.43, out of a 0–10 scale) while the average trust score was 3.72 (SD = 0.59, from a Likert scale with four levels 1–4). Only 4 participants reported having experienced in the past the actual procedure (gallbladder removal surgery).

The intervention group performed higher on the knowledge check by an average of one point when compared to the control group (p = 0.0480). Overall, questions had a correct response rate of 70–95% with the exception of question 9 “Diarrhea or altered stool consistency or color is common after gallbladder removal surgery” where only 15% of the total participants got the answer correctly. Even within this question, the teach-back method group performed significantly better whereas those who responded to the question correctly were all in the teach-back methods group. (5 out of 5, P = 0.0445). Subjective evaluation of the physician and encounter was significant for increased trust score (p = 0.0458). The length of the visits with the intervention teach-back intervention took an average of 2.45 min (147.27 s) longer than the control group visits (p = 0.0014). People who had the actual procedure in the past (evaluated as a confounder) were not significantly more likely to display the same effect as the teach-back method, suggesting that the knowledge and trust gained were not based on previous experiences with the procedure (p = 0.1946 and 0.5424 respectively) (Table 4).

**Table 4:** Effect estimates and p-values of Total score, trust Score, and timing associations. Associations were evaluated for study treatments (Teach-Back method vs. Control) and the confounder (Had actual procedure in the past No vs. Yes) Standard errors are displayed in parentheses after each estimate

	Study treatment			Confounder (Had actual procedure in the past)		
Parameter	“Teach-back” Estimate	“Control” Estimate	P-value	“No” Estimate	“Yes” Estimate	P-Value
Total Score (based on total number of correct answers)	8.35 (0.33)	7.38 (0.34)	0.0480	8 (0.26)	7 (0.71)	0.1946
Trust Score (based on mean Likert score)	3.92 (0.14)	3.51 (0.14)	0.0458	3.74 (0.11)	3.55 (0.30)	0.5424
Timing (seconds)	529.5 (29.2)	382.3 (30.1)	0.0014	445.9 (25.7)	546.5 (69.2)	0.1827

## Discussion

The results of this study are consistent with previously conducted studies on the teach-back method in other medical specialties, as well as those few that study its use in surgery. Namely, Fink et al. [11], in a 2010 study, tested an electronic variation of teach-back in the informed consent process and showed an increase in total mean comprehension scores. However, trust and time were not measured [11]. A follow-up to this study conducted by Prochanzka et al. in 2014 showed that surgical patients were highly satisfied with teach-back during the informed consent process, that teach-back did not deter from the process, and that teach-back improves informed consent, which is also consistent with our findings [12].

The potentially life-altering nature of surgical therapy most definitely shapes the relationship between the surgeon and his or her patient. This demands an immense amount of trust from the patient. Surgical patients for a time give up complete control of their care, trusting their surgeon to perform and act in such a way that had been discussed prior. Medical patients, in comparison, usually retain a substantial degree of control over their care. Therefore, emphasis must be placed on the initial trust that is built between a surgeon and the patient [15]. As was shown in our study, an added average of 2.45 extra minutes spent interacting with patients using this method can have significant improvements in patient-physician trust and patient involvement with shared decision-making. Surgeons and all physicians alike can improve the life and health of their patients by taking the time to cultivate trust rather than merely trying to do so by achieving technical successes.

### Informed consent

is more than just the signing of a form. It is a thorough and thoughtful process of communication between a patient and provider. The American College of Surgeons states informed consent as, “presenting information fairly, clearly, accurately and compassionately” [16]. Furthermore, in a climate that is becoming increasingly litigative, teach-back has the potential to protect the surgeon by optimizing the informed consent process by checking for understanding. To ensure teach-back is well received, it is important to emphasize that you are not quizzing patients on their health literacy. Rather, it is better to assert you are re-enforcing your ability to communicate imperative information.

This study has some limitations. Our limited sample size of 34 patients in a simulation-based setting did show compelling evidence that needs to next be replicated in actual medical scenarios with large groups of surgical patients. Additionally, in this study, participants were not experiencing the reality of anxiety, pain, and other emotions that accompany many patients before surgical procedures, despite our best efforts to limit confounding variables by simulating realistic pre-operative encounters. Another limitation of our study is that all standardized patients and all student participants spoke English as their primary language, potentially masking the effect of language barriers that may avert the successful application of the teach-back method when the primary languages of physician and patient do not match.

The necessity for a scale in the answer choices for our survey adds some subjectivity to the results. Our approach reveals a significant increase as a result of the implementation of the teach-back method. However, we are limited by nonexistent context to effectively interpret the objective assessment of information retention, confidence, and trust. Our study pioneers in describing this type of assessment so there are no reference points to compare. Additionally, we do not know what the average baseline might be for information retention, much less trust. Future investigations are needed to follow up on this concept to define this scale and to motivate others to cross-validate our findings.

To further support the significant findings of this study, future investigations should be performed. Replicating the methods and procedures of this study in an actual surgical setting with real surgeons and patients on a larger scale will offer further insight. Ideally, optimal validity would likely be found in a clinical practice involving multiple surgical subspecialties conducting a variety of surgical procedures with varying degrees of morbidity and mortality. Additionally, trends could be analyzed by comparing the benefits of the teach-back method in surgical candidates undergoing elective compared to urgent procedures, modifying the technique to accommodate time constraints specific to each scenario. The effect of implementing teach-back while utilizing a professional medical interpreter should be further investigated.

Studies involving this pre-operative method could also be employed in a pediatric setting exploring the complexities of application among both children of varying ages and their parents prior to surgery. Cross-language implementation of the teach-back method could be investigated using credentialed interpreters to explore the complexities and perceived benefits involved with English-speaking physicians treating patients speaking a variety of languages using this application of informed consent. Additionally, the recent surge in clinical research exploring implicit biases among physicians towards patients of different races and cultural backgrounds could be further explored in the context of the teach-back method application to improve culturally competent and unbiased care.

The methods shown in this study to improve shared decision-making, comprehension, and surgeon trust can be easily implemented at minimal time cost to practicing physicians. If future studies can find significant evidence supporting our findings in a clinical setting with real patients, the recommendation of the teach-back method to be employed before all surgical procedures may be appropriate. Withholding these potential benefits from patients backed by strong supporting evidence without reasonable cause prior to their invasive medical procedures might deviate from the optimal standard of care.

A final point to consider is whether or not there is a patient-driven demand for improved informed consent and shared decision-making. Further inquiry into whether or not a significant number of patients are currently unsatisfied with the quality of their preoperative encounters with their surgeons should be conducted. It would be important to understand if the vast majority of patients prefer to entrust the correction of their varying maladies to their surgeons without feeling the need for elevated comprehension, trust, and shared decision-making. Our findings of increased trust perception among our standardized patients suggest there may be a demand.

## Conclusion

This proof-of-concept study proved to a degree that the teach-back method is effective in the perioperative informed consent discussions in both enhancing patient comprehension of risks and benefits and generating surgeon trust. Incorporating the teach-back method into risk and benefit disclosures effectively informs and more fully engages patients in the informed consent process. Notably, the added benefits from using teach-back can be obtained without a burdensome increase in the length of visit. Increased patient knowledge improves informed consent and benefits both the patient and the doctor. Implementing teach-back in pre-operative discussions may improve shared decision-making in the informed consent process, and its use in the preoperative informed consent process should be further evaluated in larger-scale studies conducted in the surgical setting to evaluate how to optimize its implementation.

## Data Availability

Data set is not published but is available upon request.
